# Parietal and peritoneal localizations of hepatocellular carcinoma: is there a place for a curative surgery?

**DOI:** 10.1186/1477-7819-12-298

**Published:** 2014-09-25

**Authors:** Nazario Portolani, Gian Luca Baiocchi, Federico Gheza, Sarah Molfino, Daniele Lomiento, Stefano Maria Giulini

**Affiliations:** Department of Clinical and Experimental Sciences, Surgical Clinic, University of Brescia, Brescia, Italy

**Keywords:** Liver, HCC, Recurrence, Peritoneum, Parietal wall, Prognosis, Surgery

## Abstract

**Background:**

The clinical course of peritoneal and parietal recurrence of hepatocellular carcinoma (HCC-PPL) is not well known.

**Methods:**

Twenty-eight patients with a histologically proven HCC-PPL were analyzed out of a series of 515 patients operated for HCC (group 1). The risk factors, histological features, growing dynamic and results of surgical treatment were analyzed and compared with patients having other extrahepatic localizations of HCC (group 2; 26 patients). Survival data were also compared with patients with intrahepatic-only recurrence (group 3; 211 patients).

**Results:**

In group 1, a needle tract injury was present in 57.1% and a previous spontaneous rupture in 14.3% of cases. Parietal seeding was generally single, while peritoneal seeding was frequently multiple. Grading was poor in 84.7%, microvascular infiltration was observed in 57.1% and a rapid growth in 55.5% of cases. In Group 2, only 4 out of 26 patients underwent surgery. Survival was significantly better in group 3 than in group 1, and in group 1 than in group 2.

**Conclusions:**

Extrahepatic HCC recurrence is related to an aggressive biology of the cancer; many characteristics of high malignancy are usually present in these cases. After radical surgery for HCC-PPL, an acceptable survival may be obtained.

## Background

In patients with hepatocellular carcinoma (HCC), extrahepatic recurrences are generally considered as an expression of an advanced cancer not suitable for surgery [1–3]. Among these localizations, the growth of cancer in the abdominal or thoracic wall and in the peritoneal cavity is often regarded as the result of a neoplastic seeding, induced by the passive transportation of tumor cells out of the liver [4]. Some kind of “violation” of HCC integrity is often demonstrated in these cases and interpreted as a possible pathogenetic event. A percutaneous procedure (needle tract injury), including needle biopsy (PB), ethanol injection (PEI) or radiofrequency ablation (RFA), is generally recorded [5]. Some cases are reported also after transarterial chemoembolization and after surgery, as a consequence of necrosis and intraoperative manipulation, respectively. Finally, a spontaneous rupture of HCC may produce a spillage of tumor cells in the peritoneal surface [6, 7] with possible seeding and multiple nodule growth.

It is not clear if the parietal and/or peritoneal localizations of HCC (HCC-PPL) present the same biologic and prognostic value as other extrahepatic metastases - simply representing the growth of viable cells outside the liver - or, on the contrary, if they are related to a particular biologic aggressiveness of the neoplasm. Literature is very scarce in this field [6, 8, 9]; in the published studies, HCC-PPL is generally considered together with other extrahepatic localizations [10, 11]. Furthermore, the largest surveys and few multicentric reviews available are oriented toward the risk of seeding after the different percutaneous procedures rather than toward the descriptions of the natural history of these patients [12–14].

In this study, we report a series of patients with HCC-PPL and discuss the pathogenesis, clinical manifestation, treatment and survival in the light of our 20 years experience of more than 500 patients submitted to liver resection for HCC.

## Methods

This retrospective study includes, out of 515 HCC patients operated in the period 1990 to 2011 in the Surgical Clinic of the University of Brescia, 28 patients with HCC-PPL (group 1), 26 patients with extrahepatic recurrence of HCC not located in the thoracic and abdominal wall nor in the peritoneum (group 2) and, only for survival analysis, 211 patients with intrahepatic-limited recurrence (group 3). Recurrences were always diagnosed by computed tomography (CT) or magnetic resonance imaging; PB of the recurrence was performed in selected cases. A needle tract injury was considered as the causative event of PPL if there was an exact correspondence of the parietal seeding with the trajectory of the needle. Delay of appearance was noted, together with the pathological features (location, size, number, presence of a capsule, microvascular infiltration, invasion of the surrounding tissue) both for the primary HCC and of HCC-PPL. The rate of growth of HCC-PPL was assessed by comparing CT to the final size on the pathology report. Surgical resection was attempted in every patient with an extrahepatic disease, provided that the surgical risk was acceptable and that a radical resection could be attempted, with the exception of bone metastases which were treated with radiotherapy. Time and type of a further recurrence, the need of an iterative surgery and the reason for death were also recorded.

Overall survival, both from the treatment of the primary tumor and from the treatment of recurrence, was determined and compared between the three groups.

### Statistical analysis

The variables were scheduled as discrete or continuous, and comparison was performed by means of the χ^2^ test and the Student *t* test, respectively. Survival rates were calculated using the Kaplan-Meier method; comparison between the curves was performed by means of the Cox proportional hazards model. *P* values less than 0.05 were considered statistically significant.

## Results

### Study group

**Figure 1:**
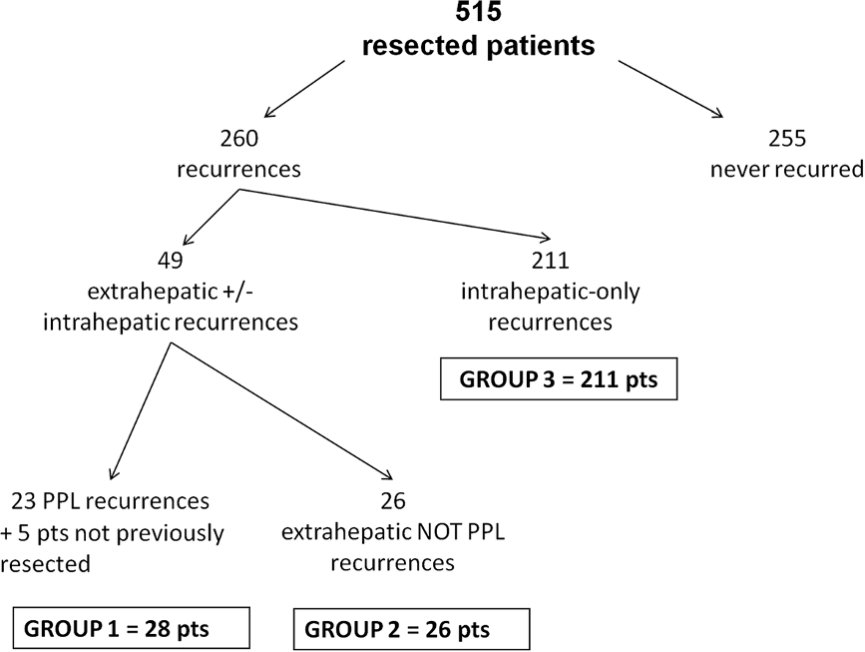
**Flow chart of patient’s selection.** PPL, parietal and/or peritoneal localization; pts, patients.

Cancer recurrence was diagnosed in 260 out of 515 patients resected for HCC (50.5%); in the majority of cases (211 patients), the recurrence was only intrahepatic. The remaining 49 patients showed an extrahepatic recurrence (multiple localizations in 34 cases). In 28 patients (5.4%), the extrahepatic recurrence involved the thoracic or the abdominal wall (13 patients) and/or the peritoneum (17 patients). Two patients had both parietal and peritoneal recurrence. In 26 cases an extrahepatic recurrence not involving parietal wall and peritoneum was observed (group 2). Study groups are summarized in Figure 1. In both groups 1 and 2, in more than half of cases the extrahepatic recurrence was associated with a synchronous liver recurrence.

### Pathogenesis, clinical and pathological data

Clinical and pathological data are shown in Table 1. In group 1, the primary HCC was exophitic in 39.3% of cases, and the mean size was 6.2 cm (range 1 to 25 cm). A needle tract injury was present in 15 patients (53.6%), 10 patients being submitted to a PB before surgery and the other five patients being treated with PEI or RFA as a primary therapy. Another four patients developed peritoneal localizations after resection of ruptured HCC and one patient after transarterial chemoembolization. Thus, eight patients (28.6%) showed an HCC-PPL without iatrogenic or spontaneous tumor rupture. Primary HCC of group 2 patients was similar in localization, while no previous PB nor PEI/RFA were recorded in group 2.

**Table 1:** **Clinical and pathological data of group 1 and group 2 patients**

Parameter			Group 1 (n = 28)	Group 2 (n = 26)
Primary HCC	Site	Exophitic	39.3%	30.8%
		Subcapsular	7.1%	11.5%
		Deep	53.6%	57.7%
	Primary treatment	Resection	82.1%	100%
		PEI/RFA	17.9%	0%
	Tumor violation	Needle tract injury	53.6%	7.7
		Spontaneous HCC rupture	14.3%	0%
		TACE	3.6%	0%
		No violation	28.6%	92.3%
Number of PPL/extrahepatic lesions		Single	53.6%	0%
		Multiple	46.4%	100%
Intrahepatic-associated recurrences			60.7%	53.8%
Delay of appearance of recurrence after the primary treatment (months)	After resection	Mean	20	13
		Range	1-45	2-44
	After PB/PEI/RFA	Mean	15	–
		Range	4-23	–

HCC, hepatocellular carcinoma; PB, percutaneous biopsy; PEI, percutaneous ethanol injection; PPL, parietal and/or peritoneal localization; RFA, radiofrequency ablation; TACE, transarterial chemoembolization.

Seeding was single in 15 patients and multiple in 13 patients (range 2 to 20 nodules) in group 1; the relative incidence of single seeding varied according to the localization of HCC implantation (100% for parietal localization and 11.7% for peritoneal site; *P* < 0.001) and the causative mechanism (50% after needle injury and 0% after HCC rupture; *P* = 0.07). On the contrary, extrahepatic recurrence was always multiple in group 2.

HCC-PPL appeared with a delay of 20 months on average, while non-PPL recurrence appeared with a 13-month delay (*P* = not significant); the delay of PPL implantation was different according to the causative mechanisms (24 months after needle tract injury, 8 months after spontaneous or iatrogenic HCC rupture, and 26 months when no clear causative mechanism was present). Delay was 15 months on average (range 4 to 23) in the five patients primarily treated with PEI/RFA. The diagnosis of metachronous HCC-PPL was made at first by clinical evaluation (seven cases of parietal seeding) or CT during follow-up. At CT, the lesions appeared as nodules in the parietal wall or in the peritoneal cavity without ascites or peritoneal thickening.

### Treatment of patients presenting with parietal and/or peritoneal localizations of hepatocellular carcinoma or other extrahepatic recurrences

All 28 HCC-PPL patients were classified as “Child A” (Child-Pugh Score). Surgery was attempted for all cases and carried out in all but one (presenting multiple peritoneal and hepatic recurrences; after open biopsy, this patient was treated with supportive care and died in 1 month).

For the treatment of parietal localization of HCC (13 patients), simple excision of the nodule was performed in two patients and *en bloc* resection of the abdominal/thoracic wall in 11 patients (two of these patients needed resection of the thoracic wall with the removal of the rib and a reconstructive plastic surgery. For peritoneal metastases, resection was limited to the lesions in seven cases and extended to the bowel, the stomach and the liver for a direct infiltration in the other six; one patient with peritoneal seeding of more than 20 nodules after laparoscopic RFA of a small HCC required the resection of the right kidney, the transverse colon and the small bowel (Table 2). The synchronous liver recurrence was treated in 15 patients by liver resection and in one patient by PEI. Mean postoperative complications were pleuric effusion (11.1%), ascites (11.1%), and anemia (3.7%) as summarized in Table 2. Mortality was nil; mean hospital stay was 8 days.

**Table 2:** **Surgical procedures and postoperative complications of group 1**

Treatment of parietal localization of hepatocellular carcinoma	Number of patients	Postoperative complications
Simple excision of the nodule	2	–
*En bloc* resection	11	2 pleural effusion, 1 ascites
Resection limited to the peritoneal nodules	7	1 ascites
Resection extended to bowel, stomach and liver	6	1 pleural effusion, 1 ascites
Resection of kidney, transverse colon and small bowel	1	1 anemia

In the 26 patients with extrahepatic recurrences without HCC-PPL, surgery was feasible only in four cases (15.4%), including a solitary metastasis in the lung, adrenal gland, rib and lymph node at the hepatic pedicle. In five further patients, bone metastases were treated with radiotherapy; one patient received treatment with Sorafenib.

### Histological features

In group 1, 83 lesions were completely removed (free surgical margin in all cases) including 30 nodules missed at CT in six patients. The size was 4.8 cm on average (range 1 to –22 cm), being larger than the primary HCC in five cases. A significant increase (>25% in size) was noted in five out of nine patients operated on 1 month after CT. Grading was G1 in four cases and G2-G3 in 24 cases; all the multiple lesions showed the same differentiation. In six patients, grading was poorer than the primary HCC. Microvascular infiltration was noted in 16 cases. The lesion infiltrated the surrounding tissue in 3 out of 13 parietal and 8 out of 15 peritoneal localizations. All eight patients without needle tract injury or HCC rupture presented in the primary HCC a poor grading and a capsule infiltration histologically proven. Primary HCC was beyond Milan criteria in seven out of these eight patients; two patients had lymph node metastases at the time of primary surgery (Table 3).

**Table 3:** **Macroscopic and histological data extrahepatic recurrence of HCC**

Tumor characteristics		Group 1 (n = 28)	Group 2 vn = 26)	***P***
Size	≤3 cm	60.7%	11.5	<0,001
	>3 cm	39.3%	88.5	
	Mean (range) (cm)	4.8 (1-22)	8.1 (1.5-16)	
Grading	G1	10.7%	30.8	0.15
	G2-G3	89.3%	69.2	
	G > primary HCC (n)	6	2	
Capsule	Yes	28.6%	34.6%	0.63
	No	71.4%	65.4%	
Vascular infiltration	Yes	53.6%	76.9%	0.12
	No	46.4%	23.1%	
Infiltration of the surrounding organs	Yes	39.3%	65.4%	<0.031
	No	60.7%	34.6%	

Group 1, parietal and/or peritoneal localizations of hepatocellular carcinoma (HCC); Group 2, extrahepatic non-parietal and/or peritoneal recurrences.

Compared with group 1, group 2 patients had a significantly larger lesion (medium size 8.1 cm, range 1.5 to 16; *P* < 0.001) and a more frequent infiltration of the surrounding organs (*P* < 0.031) (Table 3).

### Survival data

**Figure 2:**
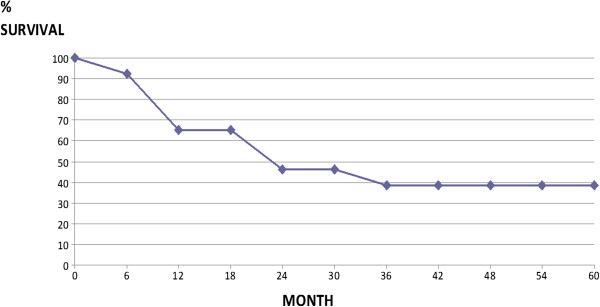
**Overall survival in 27 patients treated with surgery for parietal and/or peritoneal localization recurrences of hepatocellular carcinoma from the treatment of recurrence.**

**Figure 3:**
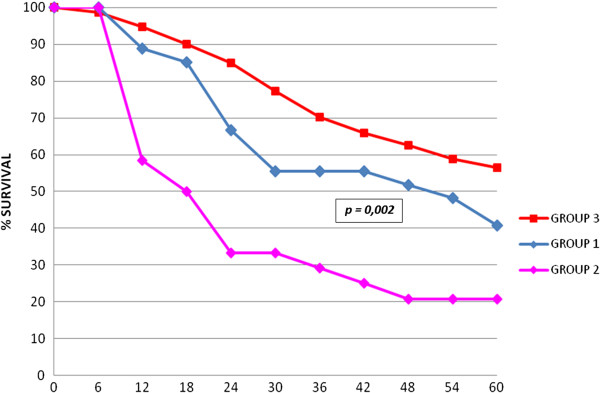
**Overall survival of patients with parietal and/or peritoneal localization (PPL) recurrences of hepatocellular carcinoma (Group 1), patients with non-PPL extrahepatic metastases (Group 2) and patients with intrahepatic-only recurrence (Group 3) after treatment of primary hepatocarcinoma.**

At the time of writing, 10 patients out of 28 (group 1) are living; death was due to liver failure in four patients and to cancer progression in 14 patients. Survival rates after surgery for recurrence were 66.7%, 44.4%, 37.0% and 37.0% at 1, 2, 3 and 5 years, respectively, with a mean survival of 16.1 months (Figure 2). Respective survival rates were 72.7%, 54.5%, 45.5% and 45.5% in the 11 patients with only HCC-PPL recurrence, and 62.5%, 37.5%, 31.3% and 31.3% in the 17 patients who presented associated hepatic recurrence (*P* = 0.21). Survival was slightly poorer in the group of 20 patients resected after iatrogenic or spontaneous HCC rupture than in the eight patients without cancer violation (5 year survival rates of 36.8% and 50.0%, respectively). Moreover, the small subgroup of patients having had a tumor rupture showed the worse results: a very low mean recurrence timing (8 versus 20 months) and a bad overall survival (15.3 versus 36 months from the primary treatment). Starting from the time of the treatment of the primary HCC, survival rates were 88.5%, 66.7%, 55.5% and 40.7% at 1, 2, 3 and 5 years, significantly better than group 2 patients (58.3%, 33.3%, 29.2% and 20.8%; *P* = 0.002), and significantly worse than group 3 patients (95.4%, 84.3%, 70.9%, 58.2%) (Figure 3). In group 2, survival after surgery (mean 15 months) was better than survival with other therapies (10 months) (*P* = 0.07) and comparable to the patients with HCC-PPL treated by surgery.

As a whole, seven patients suffered a further HCC-PPL recurrence. No local recurrence was observed after resection of a parietal seeding; in one patient, after the removal of a 2-cm thoracic wall seeding after PB of a lung metastasis, a further resection of a 1.5 cm seeding at the abdominal wall after PB of a liver recurrence was needed. After surgery for a peritoneal localization, a recurrence in the abdominal cavity was observed in six patients (mean delay 9 months), treated by surgery in three cases.

## Discussion

Our experience, the largest from a western country, suggests that HCC-PPL is rare. The main pathogenetic mechanism seems to be related to the preoperative violation of the cancer integrity rather than to an intraoperative dissemination; a needle tract injury or a spontaneous rupture of the tumor was documented in 71.4% of our cases. This hypothesis is easy to demonstrate in the parietal localization with the exact coincidence of the lesion with the trajectory of the needle, while it is not proven that percutaneous procedures or HCC rupture carry a significant increase of the risk of peritoneal localization as well [5].

Yeh and Chen observed that the rate of a spontaneous rupture was comparable between patients with peritoneal localization and patients who never recurred (25.5% and 16.4%) [8]. Kwak and colleagues, comparing the patients with peritoneal localization to a randomly matched control group, noted the same rate of needle tract injury (30.9% and 26.5%) while HCC rupture (19.1% and 4.4%) was confirmed as an independent risk factor (*P* = 0.008) [5]. These studies stated that a peritoneal recurrence does not necessarily appear in every patient after a spontaneous rupture of HCC, as in our experience too, but were unable to define the pathogenetic role of the percutaneous procedures. In an extensive literature review, including no series presenting more than 15 cases, Stigliano and colleagues summarized a median rate of seeding of 2.29% after PB and 1.4% after PEI, the lowest risk being reported for RFA (0.61%) [14]; however, a higher incidence, up to 5.1%, has been recently reported [13, 15].

The rate of growth of these lesions, accurately represented by CT [12, 16], can be particularly quick, as in 55.5% of our patients, leading to a recurrent cancer larger than the primary one in spite of the periodic controls. The analysis of the specimens showed a G2-G3 differentiation in the majority of our patients (86.6%), grading being poorer than the primary tumor in six patients. Both these data support the hypothesis that seeding could reflect a particular aggressiveness of the cancer. This fact could explain the relatively unsatisfactory results obtained by apparently radical treatments [13]. Thus, the growth of a mass in the peritoneum could be really considered as an extrahepatic metastasis; the biologic features of primary HCC in these cases were unfavorable with poor grading and infiltration of the tumor capsule.

Nonetheless, unlike the other types of peritoneal carcinomatosis, the peritoneal localization of HCC is the reason of death only in a limited number of patients [17]; the lesion is generally suitable for curative resection for the nodular aspect of the recurrence without “carcinomatosis peritonei” [6, 16]. Some patients can require an extension of resection to the nearest organs, as in 56% of our patients.

On the whole, the prognosis for these patients is not satisfactory. Our experience presents less favorable results than Ding and colleagues (2-year’s survival of 71% in a collective review of 24 patients) [18]. Nonetheless, also from this experience, survival is clearly better than for the other extrahepatic recurrences, which are submitted to a curative procedure only in a minority of cases. So, for HCC-PPL, treatment must always be considered in patients are fit for surgery, provided that other localizations are absent or suitable for radical care. At surgery, the entire abdominal space must be explored, as many small seedings could be undetected before surgery; a further peritoneal recurrence is not rare (50% in our experience), and is suitable for a further resection in selected cases [18, 19]. On the contrary, surgery for parietal localization usually does not involve the risk of further local recurrence provided that a wide *en bloc* resection of the wall is performed, extended to the surrounding organs if required [20–22].

The different mechanisms of the seeding between the peritoneal localization (violation of the tumor in open space) and the wall localization (transport of viable cells along the narrow needle trajectory) may explain the different risk of failure, even if a negative surgical margin was obtained. An aggressive attitude is justified in the peritoneal localizations resulting from seeding after spontaneous rupture of HCC as well; long-term results are acceptable, particularly considering the negative factors of these cancers at presentation.

Moreover, at this time there is no evidence in the literature for different therapies (such as radiotherapy or drugs such as Sorafenib) that could treat this kind of recurrence.

## Conclusion

HCC-PPL can be interpreted as a marker of an aggressive biology of the cancer. A radical treatment rarely means long-term survival but offers definitively better results than abstention from treatment. The better survival compared to the other extrahepatic localizations seems to be related to a favorable presentation, more frequently suitable for a surgical resection, rather than a different biologic behavior. Even if this study has the problems of a long study period, is retrospective in nature and has a small sample size, our results justify surgery in selected patients when a radical purpose is pursued in the general context of the HCC diffusion.

## Abbreviations

CT: computed tomography
HCC: hepatocellular carcinoma
HCC-PPL: parietal and/or peritoneal localizations of HCC
PB: percutaneous biopsy
PEI: percutaneous ethanol injection
RFA: radiofrequency ablation.
